# Prediction of COPD risk accounting for time-varying smoking exposures

**DOI:** 10.1371/journal.pone.0248535

**Published:** 2021-03-10

**Authors:** Joanne T. Chang, Rafael Meza, David T. Levy, Douglas Arenberg, Jihyoun Jeon

**Affiliations:** 1 Department of Epidemiology, School of Public Health, University of Michigan, Ann Arbor, Michigan, United States of America; 2 Department of Oncology, Georgetown Lombardi Comprehensive Cancer Center, Washington D.C., DC, United States of America; 3 Division of Pulmonary and Critical Medicine, Department of Internal Medicine, University of Michigan Medical School, Ann Arbor, Michigan, United States of America; Medical University of South Carolina, UNITED STATES

## Abstract

**Rationale:**

Chronic Obstructive Pulmonary Disease (COPD) is the fourth leading cause of death in the United States. Studies have primarily assessed the relationship between smoking on COPD risk focusing on summary measures, like smoking status.

**Objective:**

Develop a COPD risk prediction model incorporating individual time-varying smoking exposures.

**Methods:**

The Nurses’ Health Study (N = 86,711) and the Health Professionals Follow-up Study (N = 39,817) data was used to develop a COPD risk prediction model. Data was randomly split in 50–50 samples for model building and validation. Cox regression with time-varying covariates was used to assess the association between smoking duration, intensity and year-since-quit and self-reported COPD diagnosis incidence. We evaluated the model calibration as well as discriminatory accuracy via the Area Under the receiver operating characteristic Curve (AUC). We computed 6-year risk of COPD incidence given various individual smoking scenarios.

**Results:**

Smoking duration, year-since-quit (if former smokers), sex, and interaction of sex and smoking duration are significantly associated with the incidence of diagnosed COPD. The model that incorporated time-varying smoking variables yielded higher AUCs compared to models using only pack-years. The AUCs for the model were 0.80 (95% CI: 0.74–0.86) and 0.73 (95% CI: 0.70–0.77) for males and females, respectively.

**Conclusions:**

Utilizing detailed smoking pattern information, the model predicts COPD risk with better accuracy than models based on only smoking summary measures. It might serve as a tool for early detection programs by identifying individuals at high-risk for COPD.

## Introduction

Chronic Obstructive Pulmonary Disease (COPD) is one of the leading causes of death globally and domestically. In 2016, COPD ranked fourth, accounting 5.6% of deaths after cardiovascular diseases, cancer, and accidents in the United States (U.S.) [1]. The 2018 World Health Organization Report of Monitoring Health for the Sustainable Development Goals states that respiratory conditions, including COPD accounting for 9% of deaths globally in 2016 [2].

Cigarette smoking is the most important risk factor for COPD [3]. In the U.S., approximately 80% of COPD deaths are linked to smoking, and 20% of smokers are expected to be diagnosed with COPD [4]. In 2011, the age-adjusted COPD prevalence was 14.1% among current smokers, 7.1% among former smokers, and 2.9% among never smokers [5]. Other risk factors include age, sex, race, occupation, education, alpha-1 anti-trypsin deficiency, asthma, and exposures to other chemical fumes and air pollution [6–11].

Although numerous studies have established the association between smoking and COPD, these studies [6,12–16] have used limited smoking information (e.g., smoking status) in their analyses. Other smoking information, such as duration, intensity, and year-since-quit for former smokers, may play an important role in determining COPD risk. Furthermore, smokers could change their smoking behaviors throughout their lifetime, and these changes may affect individual COPD risk with age. Overall, better information is needed on how individual smoking histories shape age-specific COPD risk [17].

Using large prospective cohort data, we developed a COPD risk prediction model accounting for multiple time-varying smoking covariates and estimated the time-dependent effect of pack-years of smoking on the incidence of diagnosed COPD while adjusting for smoking duration, year-since-quit, age, and sex. We evaluated the model performance in terms of calibration and discriminatory accuracy and used the model to investigate how COPD risk changes as a function of smoking duration, intensity, and age.

## Methods

### Study population

The Nurses’ Health Study (NHS) [18] was established in 1976 with 121,700 female U.S. nurses aged between 30 and 55 years who responded to mailed questionnaires. The participants were asked questions about their exposures to various risk factors and medical histories, and follow-up questionnaires were sent every 2 years to update this information. In parallel, the Health Professionals Follow-Up Study (HPFS) [19] was established in 1986 with 51,529 male U.S. health professionals aged between 40 and 75 years who also received similar questionnaires. The response rate was at least 90% for each two-year cycle for both NHS and HPFS [19,20]. Although there is a decade between the start of these two studies, the birth-year distributions of the two cohorts are similar (e.g., median birth-year was 1933 for HPFS and 1934 for NHS).

The NHS participants reported any previous diagnoses of COPD on the 1988–2004 and 2008 questionnaires; the HPFS participants reported on the 1998–2008 questionnaires. Self-reported COPD status was defined by receiving any affirmative response of physician-diagnosis of chronic bronchitis or emphysema. Prevalent COPD cases diagnosed before 1998 were excluded to limit the impact of recall bias on COPD incidence estimation, although this approach excludes early onset COPD cases. The final dataset consisted of 86,711 females in the NHS and 39,817 males in the HPFS. We randomly split the data into 50–50 samples and used half of the data (N = 63,279) for model-building and the other half (N = 63,249) for validation (S1 Fig). The data did not include personally identifying information and were therefore exempt from institutional review board review.

### Smoking information

At the entry of the two Studies, the NHS participants were asked to report their ages at start and quit smoking (if former smokers) and the average smoking intensity in terms of cigarettes-per-day (CPD) while they smoked. In contrast, in the HPFS cohort, participants reported average intensity for each age category (<15, 15–19, 20–29, 30–39, 40–49, 50–59, and ≥ 60 years) before the entry of study. After entering the study, each participant reported smoking status and intensity (if smokers) every two years until the end of follow-up. The smoking intensity information was collected with the following categories: 0–4, 5–14, 15–24, 25–34, 35–44, and 45+ CPD. We then assigned the mid-point of the category as the corresponding CPD for that category, i.e., 2, 9.5, 19.5, 29.5, 39.5 and 50 CPD, respectively, and calculated pack-years as a continuous variable by dividing CPD by 20 and multiplying it with smoking duration for each individual. Smoking duration and year-since-quit were coded as zeros for non-smokers. Individual smoking histories from birth to the entry of the study were constructed by applying a similar approach as in the previous literature of lung cancer incidence in these two cohorts [21].

### Cox regression model with time-varying covariates

We used a Cox proportional hazards model to estimate the relative risk of incidence of diagnosed COPD associated with time-varying smoking covariates including cumulative pack-years, duration, year-since-quit (if former smokers), with adjustment for sex. The values for these covariates changed over the course of the smokers’ lifetime. The traditional Cox proportional hazards model cannot directly account for the variations in lifetime exposure. Therefore, to account for the time-dependent nature of these smoking covariates, we coded these covariates by using annual intervals of time, (i.e., assigned the corresponding values to each year of person-time from birth to the end of follow-up). The end of follow-up was defined as whichever comes first among the following four scenarios: death, incident diagnosed COPD, lost to follow-up, end of study. These models were fitted using the “coxph” function in R (version 3.2.0).

The underlying assumption of the Cox model is that the relative risk of disease associated with a risk factor remains constant over time. This assumption often does not hold for a chronic disease, such as COPD, which tends to develop over a long period of time, and the effect of a risk factor on disease may be modified by age. Therefore, we assumed age as an effect modifier for the association between smoking exposure and COPD [22], and modeled the non-proportionality in the relative risk by including a time-dependent interaction between cumulative pack-years of smoking and age [23]; *h*(*t*) = *λ*_0_(*t*)*e^β(t)X(t)^*, where *λ*_0_(*t*) is the baseline hazard at age “t,” *β*(*t*) is a vector of regression coefficients, and *X*(*t*) represents time-dependent covariates, including duration in years, cumulative intensity in pack-years, and year-since-quit. Non-parametric natural splines with 2 degrees of freedom were chosen to model non-linear age effects for the interaction between cumulative pack-years and age [24]. We also evaluated the interaction effect between each smoking covariate and sex. As a sensitivity analysis, we also built sex-specific Cox proportional hazards models.

### Evaluation of model performance

The discriminatory accuracy of the model was evaluated by the Area Under the receiver operating characteristic Curve (AUC) based on 6-year risks at the study entry for all individuals in the validation dataset, assuming their smoking behaviors at baseline remained unchanged during the next 6 years. To compute the age-specific COPD risk probabilities, the baseline hazard, *λ*_0_(*t*), was estimated from never smokers in the data, using the “survreg” function in R [25] with assuming Log-Normal distribution. Bootstrapping with 100 iterations was used to compute 95% CIs of the AUCs, using the “pROC” package in R [25].

### Age-specific incidence and 6-year COPD risk predictions

We evaluated the relative risks of COPD comparing age-specific incidence of COPD between current and never or former smokers. By considering continuous age and exposures, our model can provide predictions of COPD incidence risk for any time-period. As an example, we computed 6-year risk of COPD incidence for selected individual smoking scenarios. We also computed this risk with adjustment for competing cause of mortality, using age-specific life-tables stratified by smoking status obtained from the Cancer Intervention and Surveillance Modeling Network [26,27] (See S1 Text and S1 Table).

## Results

### Participants’ characteristics

Tables 1 and S1 shows participants’ characteristics at baseline (year 1976 for NHS and 1986 for HPFS) for this study. In both model building and validation datasets, males have higher smoking intensity, longer smoking duration and year-since-quit compared with females conditional on smoking status. These smoking characteristics are comparable between model building and validation datasets. Since the NHS cohort started 10 years before HPFS cohort, the age at entry is roughly 10 years younger in females compared to males. About 4% of participants were diagnosed with COPD during 1998–2008 (Table 1), with about 24% to 28% of COPD cases occurs among never smokers and with median age at COPD diagnosis from 71 to 75 (S2 Table). In general, COPD was diagnosed 4 to 6 years of age earlier for current smokers versus never and former smokers.

**Table 1:** Baseline characteristics of NHS (1976) and HPFS (1986) cohorts in model building and validation datasets.

	Model building		Validation	
	HPFS (N = 19,914)	NHS (N = 43,365)	HPFS (N = 19,903)	NHS (N = 43,346)
	Median (IQR)	Median (IQR)	Median (IQR)	Median (IQR)
**Smoking intensity (pack-year)**				
Current smokers	29.00 (24.03)	21.00 (18.00)	28.50 (24.00)	21.00 (18.00)
Former smokers	15.00 (21.50)	8.50 (13.50)	15.00 (21.50)	8.00 (13.00)
**Smoking duration (year)**				
Current smokers	30.00 (14.00)	23.00 (11.00)	31.00 (15.00)	23.00 (11.00)
Former smokers	18.00 (14.00)	11.50 (12.00)	17.00 (14.00)	11.00 (12.00)
**Year-since-quit (year)**				
Former smokers	15.00 (21.00)	11.00 (9.00)	15.00 (21.00)	11.00 (9.00)
**Age at entry (year)**	52.50 (15.90)	42.10 (12.20)	52.80 (16.00)	42.20 (12.20)
COPD cases during the follow-up* **N (%)**	700 (3.52)	1,756 (4.05)	758 (3.81)	1,780 (4.11)

* COPD incidence occurred during the follow-up (1998–2008) were included in the analysis.

HPFS = Health Professionals Follow-up Study; NHS = Nurses’ Health Study; IQR = Interquartile range; COPD = Chronic Obstructive Pulmonary Disease; N = number of individuals in the data.

### Cox model with time-varying covariates

The parameter estimates for the combined model and sex-specific models are shown in Table 2. Our models include smoking intensity, smoking duration, year-since-quit, sex, interaction between sex and smoking duration, and the interaction between age and smoking intensity. The interaction between sex and smoking intensity or year-since-quit was not significant, and was excluded. The COPD risk is 1.78 times higher in females than males (95% CI: 1.56–2.04). Smoking duration is associated with a 1.02-fold increase in COPD risk per year increase (95% CI: 1.00–1.03). Although statistically significant, the effect of year-since-quit is minor. Finally, COPD risk by smoking one additional pack-year is highest at younger ages (S2 Fig).

**Table 2:** Hazard ratio (95% CI) of variables associated with COPD risk in the model building data.

	Males-only model (N = 19,914)	Females-only model (N = 43,365)	Combined model (N = 63,279)
Variable	HR^3^ (95% CI)	HR^3^ (95% CI)	HR^3^ (95% CI)
Smoking intensity (pack-years)	0.91 (0.73 to 1.15)	1.07 (0.99 to 1.15)	1.05 (0.98 to 1.12)
Smoking duration (year)	1.02 (1.01 to 1.03)	1.02 (1.01 to 1.02)	1.02 (1.01 to 1.03)
Year-since-quit (year)	0.99 (0.99 to 1.00)	0.99 (0.99 to 1.00)	0.99 (0.99 to 1.00)
ns(Age,2)1^1^*smoking intensity	1.22 (0.82 to 1.83)	0.93 (0.81 to 1.06)	0.96 (0.86 to 1.08)
ns(Age,2)2^2^*smoking intensity	1.02 (0.91 to 1.15)	0.96 (0.93 to 1.00)	0.97 (0.94 to 0.99)
Sex^4^*smoking duration			0.99 (0.99 to 1.00)
Sex^4^			1.78 (1.56 to 2.04)

^1,2^ The interaction between age and smoking intensity was modeled as a non-linear relationship using a natural spline with 2 degrees of freedom; N = number of individuals in the data

^3^ Hazard ratios; CI = confidence interval.

^4^Reference group: Males.

### Model validation and calibration

**Fig 1:**
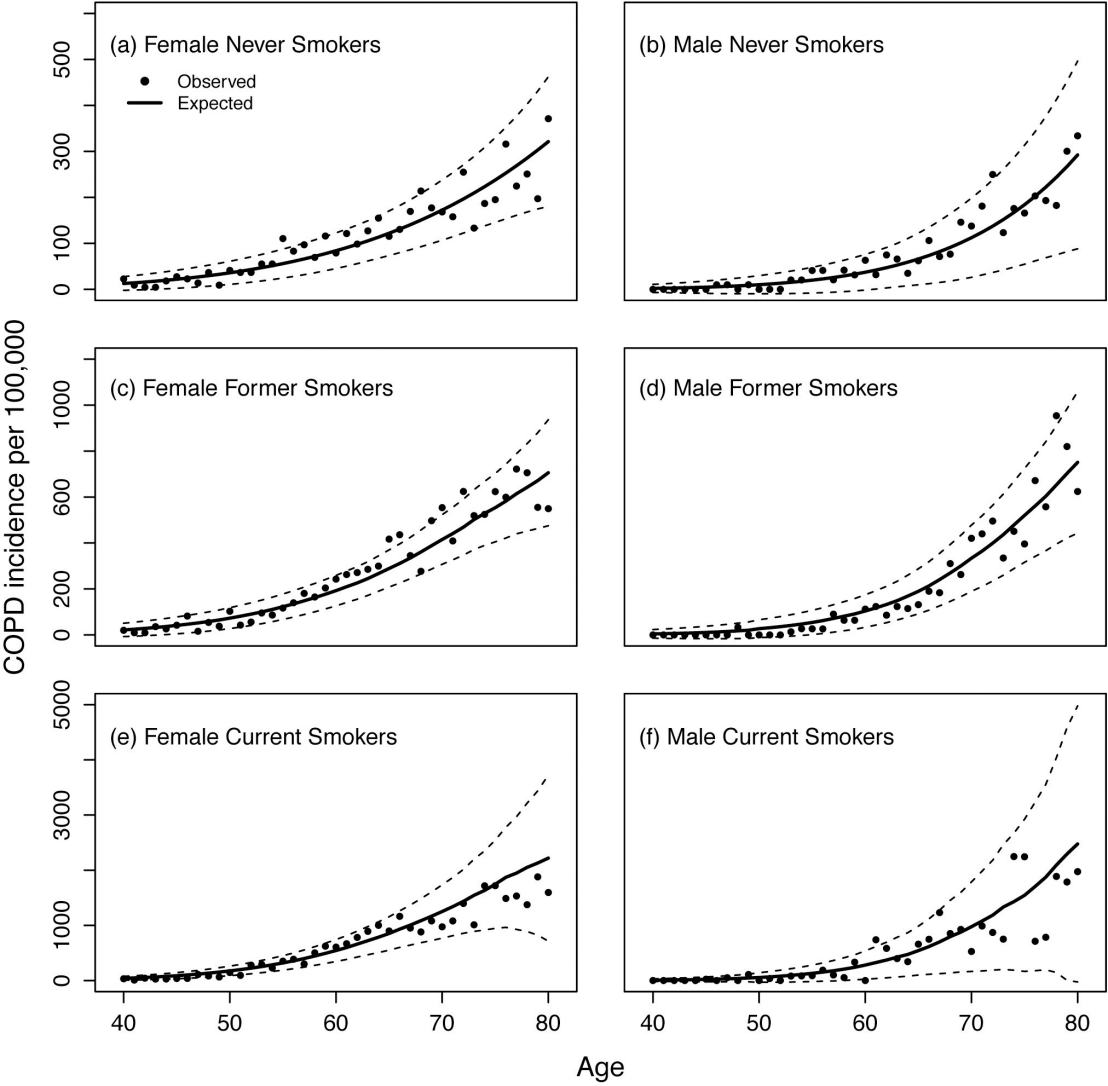
Incidence of diagnosed COPD per 100,000 for females and males by smoking status. The solid line is the expected incidence of diagnosed COPD from the model, and the dashed lines are its 95%CI. The dots represent the observed data.

Overall, our model predicts the observed incidence of diagnosed COPD well in the validation dataset. The observed incidence is within the 95% CIs of the predicted incidence by smoking status (Fig 1). A goodness of fit test shows that there is no significant difference between predicted and observed incidences in all smoking subgroups.

**Fig 2:**
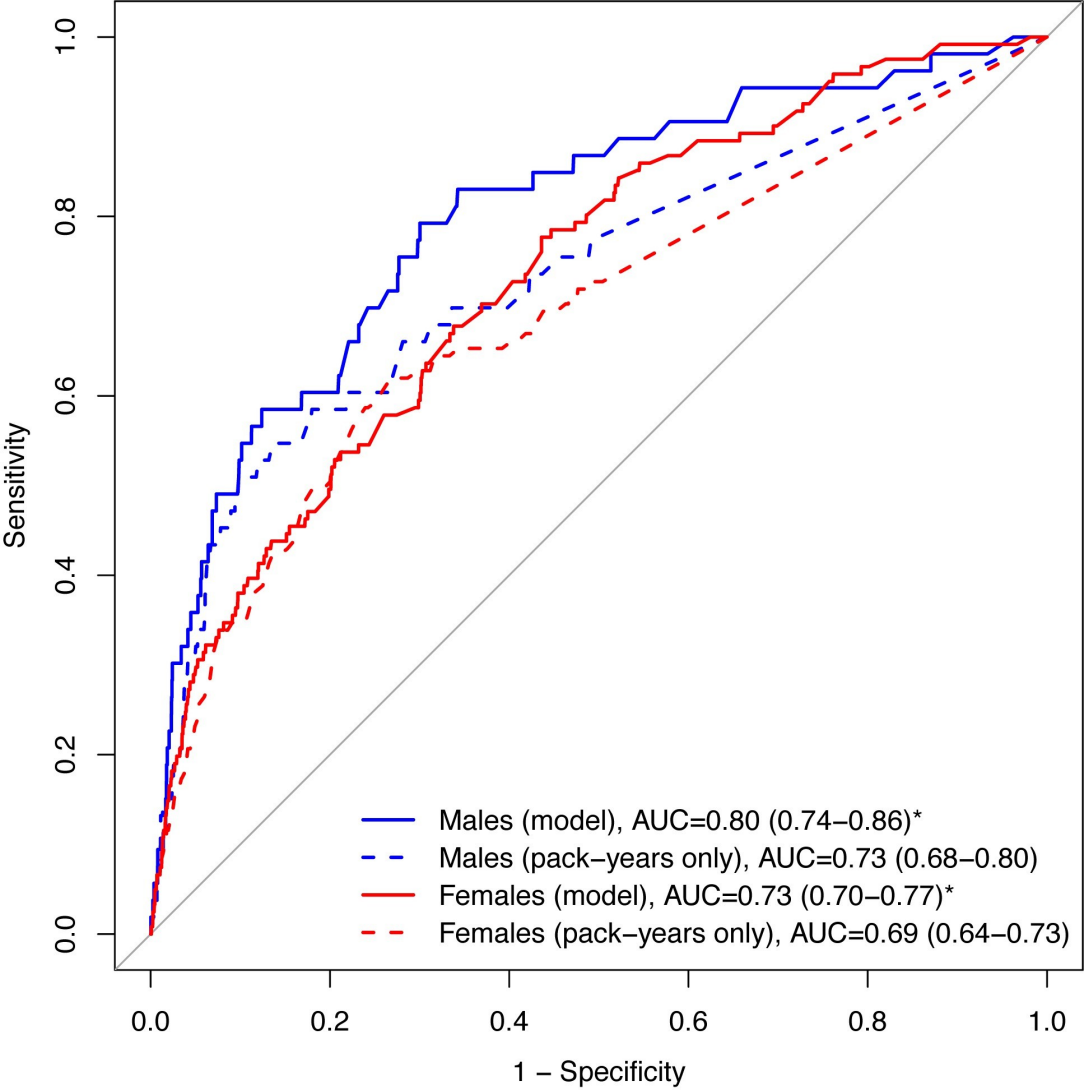
Discriminatory accuracy of models. Receiver operating characteristic curve (ROC) and the corresponding area under the receiver operating characteristic curve (AUC) comparisons between the combined model and the pack-years only model [AUC (95% CI)]. * Significantly better than pack-years only model at p<0.05.

We compared the discriminatory accuracy of our model with a model based on pack-years only (Fig 2). The AUCs for our combined model in the validation dataset were significantly higher (male: 0.80 (95% CI: 0.74–0.86); female: 0.73 (95% CI: 0.70–0.77)) than the AUCs from the pack-years only model (males: 0.73 (95% CI: 0.68–0.80); females: 0.69 (95% CI: 0.64–0.73)).

### Age-specific incidence and relative risk

**Fig 3:**
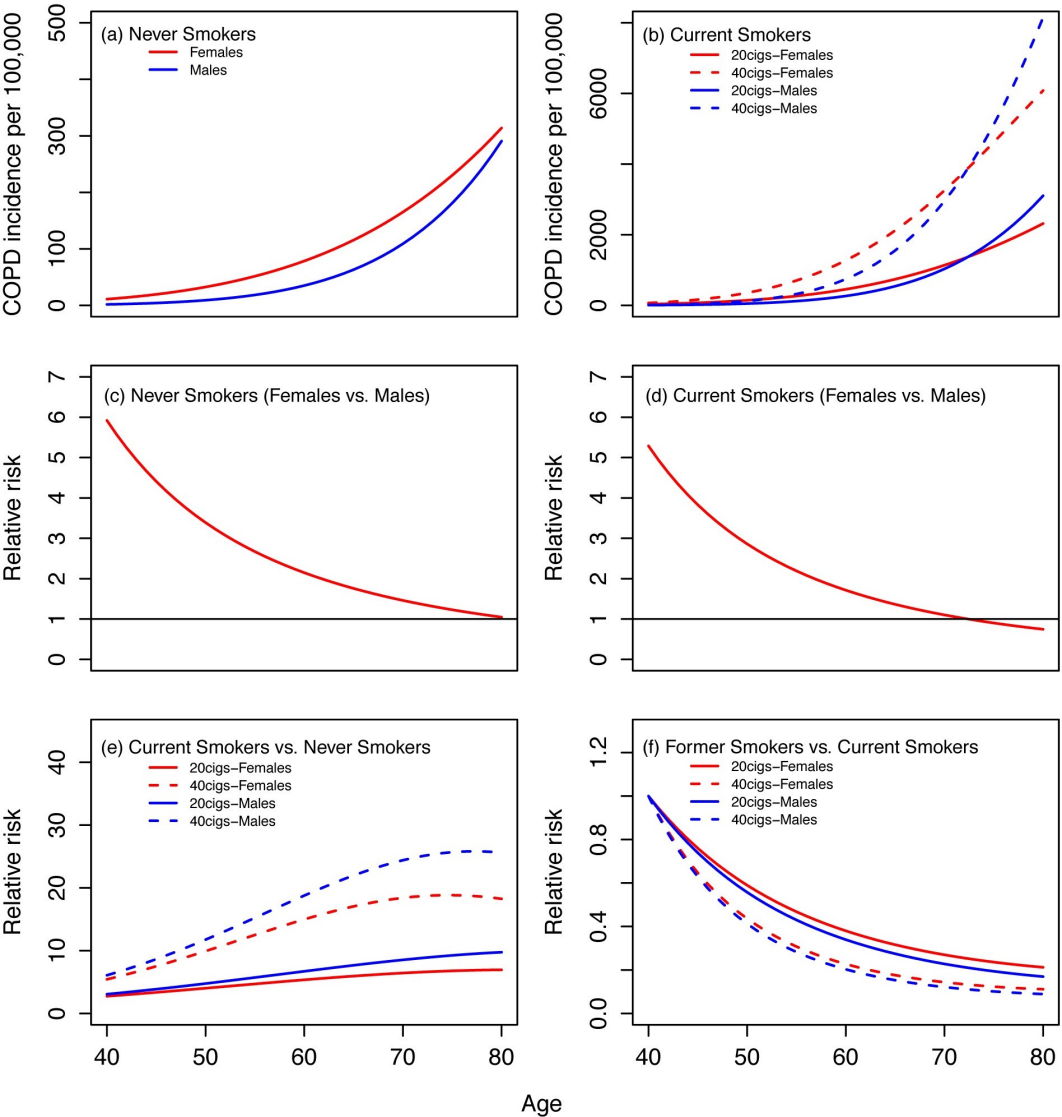
Examples for age-specific incidence rates and relative risks of COPD. (a) and (b) show the age-specific COPD incidence rates (per 100,000) among never smokers and current smokers. (c) and (d) show the relative risk of COPD of females vs. males among never smokers and current smokers (20 CPD). (e) and (f) show the relative risk of COPD of current smokers vs. never smokers and former smokers vs. current smokers, respectively. Smokers were assumed smoking either 20 or 40 CPD starting at age 20. Former smokers were assumed to quit smoking at age 40. Blue lines (males); Red lines (females).

Fig 3 shows age-specific incidence and relative risks (RR) of COPD by sex under some selected smoking scenarios. We considered smokers who smoked 20 or 40 CPD, starting at age 20 throughout their lifetime (current smokers) or from age 20 to 40 (former smokers). The top two panels in Fig 3 show the age-specific COPD incidence among never and current smokers for both males and females. For never smokers (top left panel), the baseline incidence is higher in females than males regardless of age. For current smokers (top right panel), the incidence is higher in females than males for those aged 40 to 70; however, the pattern reverses for those over age 70. The middle two panels of Fig 3 show the RR of COPD by sex, females vs. males, among never smokers (left panel) and current smokers who smoked 20 CPD, starting at age 20 (right panel). Although female never smokers have higher COPD risk than male never smokers, the difference in COPD incidence between sexes decreases when people get older. The bottom left panel of Fig 3 shows the RR of COPD of current smokers compared to never smokers. As an example, a 60-year-old female current smoker who smoked 40 CPD starting at age 20 has 15 times higher COPD risk than a never smoker at the same age. The bottom right panel of Fig 3 shows the RR of COPD of former smokers compared to continuing smokers. Former smokers have lower COPD risk relative to current smokers once they quit smoking. For example, a 60-year-old female former smoker, who smoked 40 CPD starting at age 20 but quit at age 40 has only 20% of chance of getting COPD compared to a continuing smoker at the same age.

### 6-year COPD risk predictions

Using our combined model, we computed the probability of being diagnosed with COPD in the next 6 years for selected smoking scenarios with/without accounting for other causes of death (Tables 3 and S3). For example, for a 70-year-old female current smoker who has smoked 20 pack-years over 30 years (i.e., smoked about 13 CPD for 30 years on average), the probability of COPD diagnosis in the next 6 years is 3.2% (95% CI: 3.2%-3.3%), while a 70-year-old female who has smoked 40 pack-years over the same duration has a 4.8% (95% CI: 4.7%-4.9%) risk of COPD diagnosis. If this same female quit smoking at age 70, her risk of COPD diagnosis in the following 6 years is reduced to 4.2% (95% CI: 4.1%-4.3%).

**Table 3:** Examples of 6-year absolute risk estimates for incidence of diagnosed COPD. Selected scenarios include current or former smokers at age 50, 60, 70 or 80, who have smoked either 20 pack-years or 40 pack-years. The smoking duration varies by 20, 30, or 40 years. These 6-year risk estimates were calculated without adjusting for other causes of mortality.

		Smoking Duration (years)					
		20 years		30 years		40 years	
Scenario	Age (year)	Current smokers Risk % (95% CI)	Former smokers* Risk % (95% CI)	Current smokers Risk % (95% CI)	Former smokers* Risk % (95% CI)	Current smokers Risk % (95% CI)	Former smokers* Risk % (95% CI)
**Females: 20 PY**	50	0.8 (0.8 to 0.8)	0.7 (0.7 to 0.7)	0.9 (0.9 to 0.9)	0.8 (0.8 to 0.8)	1.1 (1.0 to 1.1)	0.9 (0.9 to 1.0)
	60	1.6 (1.6 to 1.6)	1.4 (1.4 to1.4)	1.8 (1.8 to1.9)	1.6 (1.6 to1.7)	2.1 (2.0 to 2.2)	1.9 (1.8 to 1.9)
	70	2.8 (2.8 to 2.9)	2.5 (2.5 to 2.5)	3.2 (3.2 to 3.3)	2.9 (2.8 to 2.9)	3.7 (3.6 to 3.8)	3.4 (3.3 to 3.4)
	80	4.4 (4.4 to 4.5)	4.0 (3.9 to 4.0)	5.0 (4.9 to 5.1)	4.6 (4.5 to 4.7)	5.8 (5.6 to 5.9)	5.3 (5.1 to 5.4)
**Females: 40 PY**	50	1.6 (1.5 to 1.6)	1.2 (1.2 to 1.3)	1.7 (1.6 to 1.8)	1.4 (1.3 to 1.5)	1.9 (1.8 to 2.0)	1.7 (1.6 to 1.7)
	60	2.8 (2.7 to 2.9)	2.2 (2.2 to 2.3)	3.1 (3.0 to 3.2)	2.6 (2.5 to 2.7)	3.5 (3.4 to 3.6)	3.0 (2.9 to 3.1)
	70	4.4 (4.3 to 4.5)	3.6 (3.5 to 3.7)	4.8 (4.7 to 4.9)	4.2 (4.1 to 4.3)	5.5 (5.3 to 5.6)	4.8 (4.7 to 4.9)
	80	6.0 (5.9 to 6.3)	5.2 (5.1 to 5.4)	6.7 (6.5 to 7.0)	5.9 (5.8 to 6.1)	7.5 (7.3 to 7.8)	6.7 (6.6 to 6.9)
**Males: 20 PY**	50	0.3 (0.3 to 0.3)	0.3 (0.3 to 0.3)	0.4 (0.4 to 0.4)	0.3 (0.3 to 0.3)	0.5 (0.4 to 0.5)	0.4 (0.4 to0.4)
	60	1.0 (1.0 to 1.0)	0.8 (0.8 to 0.9)	1.2 (1.1 to 1.2)	1.0 (1.0 to 1.1)	1.5 (1.3 to 1.5)	1.3 (1.2 to 1.3)
	70	2.5 (2.4 to 2.6)	2.2 (2.1 to 2.2)	3.1 (2.9 to 3.2)	2.7 (2.5 to 2.7)	3.7 (3.4 to 3.9)	3.3 (3.0 to 3.4)
	80	5.4 (5.0 to 5.8)	4.7 (4.6 to 4.9)	6.5 (6.1 to 6.7)	5.8 (5.4 to 6.0)	7.8 (7.2 to 8.2)	7.0 (6.5 to 7.3)
**Males: 40 PY**	50	0.6 (0.6 to 0.7)	0.5 (0.5 to 0.5)	0.7 (0.7 to 0.8)	0.6 (0.5 to 0.6)	0.9 (0.8 to 0.9)	0.7 (0.7 to 0.8)
	60	1.7 (1.7 to 1.8)	1.4 (1.3 to 1.4)	2.0 (1.9 to 2.1)	1.7 (1.6 to 1.7)	2.5 (2.2 to 2.6)	2.1 (1.9 to 2.2)
	70	3.9 (3.8 to 4.1)	3.2 (3.0 to 3.3)	4.6 (4.3 to 4.8)	3.9 (3.7 to 4.0)	5.5 (5.0 to 5.8)	4.7 (4.4 to 4.9)
	80	7.4 (7.0 to 7.7)	6.2 (5.9 to 6.4)	8.6 (8.1 to 9.1)	7.4 (7.0 to 7.8)	10.2 (9.5 to 10.8)	8.9 (8.4 to 9.4)

*Former smokers stop smoking at the corresponding age 50, 60, 70 or 80; PY = Pack-years; CI = Confidence interval; The 95% CIs were calculated using the Bootstrap method with 100 iterations.

## Discussion

We developed a risk prediction model for the incidence of diagnosed COPD using data from the NHS and HPFS cohorts. To our knowledge, this is the first COPD risk prediction model incorporating individual time-varying smoking covariates: intensity, duration, and year-since-quit in the U.S. We found that smoking duration, intensity, year-since-quit, interaction of sex and duration, and sex were all significantly associated with COPD incidence. However, the effect of year-since-quit is relatively small compared to other factors, suggesting that the COPD risk induced by smoking is somewhat permanent. Additionally, we found the COPD risk by smoking one more pack-year is highest at younger ages. Our model validated well, has high discriminatory power, and predicts COPD risk utilizing detailed individual smoking histories.

### Relative risks of COPD by smoking status

Smoking is linked to 80% of prevalent COPD cases in the U.S. [4]. A meta-analysis found that COPD prevalence in current smokers is about 30% higher than in former smokers [17]. However, this study included only a single smoking measure (smoking status) and was unable to provide age-specific relative risks for COPD prevalence. Our model can predict the probability of being diagnosed with COPD at different ages given a person’s smoking history and showed that continued smoking is associated with increased COPD risk with different rates by age compared to former smokers.

### Year-since-quit

Our results showed that year-since-quit has a borderline beneficial effect for COPD, which could be due to several factors. First, when lifetime smokers are told that they have COPD, they may subsequently quit smoking. This change in smoking behavior shortly after the disease develops can make it seem as if former smokers are more likely to develop COPD than current smokers (reverse causation). Second, since our outcome was self-reported COPD diagnosis, it does not indicate the biological onset of COPD, in which patients might have developed COPD long before the diagnosis. Thus, if quitting occurs between incident COPD and diagnosis, it would have no effect on COPD incidence risk followed by diagnosis later since it already occurred. In contrast, quitting could lead to a false sense of no COPD risk, making it less likely that individuals would be tested clinically for airflow obstruction. Moreover, it is also plausible that the effects of smoking on the lungs that lead to COPD are non-reversible, so quitting may not decrease, but rather slow down the development of COPD.

### Comparison to previous COPD risk prediction models

The COPD incidence observed in NHS and HPFS is comparable with other cohorts [6,12,13,15,16,28]. The Rotterdam study [13] showed that the overall age-specific incidence rates of COPD per 100,000 person-years between age 60 and 70 ranged about 1,500–2,500 in current smokers, about 700–900 in former smokers, and about 300–500 in never smokers. In Fig 1, our study shows that the incidence rates per 100,000 person-years in current smokers between age 60 and 70 ranged from 544–1,251 in females and 280–975 in males. Among former smokers, the COPD incidence rates ranged from 193–415 in females and 104–334 in males; for never smokers, 84–172 in females and 37–112 in males. While the patterns are consistent (higher rates by smoking status and higher rates in females vs. males), the COPD incidence rates in NHS/HPFS are lower than the Rotterdam study.

Several studies have examined the incidence of COPD by smoking status in various populations [6,29,30]. Using data on COPD diagnosis recorded by general practitioners in Scotland, Kotz *et al*. [29] developed a COPD risk prediction model. Adjusting for deprivation index and prior asthma history, they found that the incidence of COPD is 9.61 and 6.72 times higher in ever-smokers compared to never-smokers in females and males, respectively. Gershon *et al*. [31] also found that the lifetime risk of COPD is 3.89 times higher in ever smokers compared to never smokers, adjusting for age, sex and underlying comorbidities, in Canada. Our model complements these earlier models by incorporating time-varying smoking variables and yields consistent results. In addition, we also provide estimates of the age-specific relative risk of COPD associated with increases in intensity.

### Relative risks of COPD by sex

Our analyses suggest that female never smokers have higher age-specific incidence of diagnosed COPD than male never smokers, although this finding is based on a relatively low number of COPD cases among never smokers. We also found that female smokers tend to have higher COPD risk than male smokers who have the same smoking histories at young ages, but lower risk at old ages. These sex differences could be due to multiple factors. First, health-care seeking behaviors may differ by sex, which may affect COPD diagnosis [32–34]. Our study used self-reported COPD diagnosis from participants, and some studies have shown that females are more likely to seek medical attention [35], and thus have higher rates of diagnosis. In contrast, Chapman et al. have suggested that there is a potential bias towards identification of male COPD cases [36], since physicians are more likely to refer males to spirometry due to their higher smoking prevalence than females. Second, sex-differences in risk could also be due to biological differences. For instance, females have smaller lungs than males, potentially causing more concentrated cigarette smoke exposure in a smaller volume, which may lead to higher effective exposure “per cell” [37]. In addition, some studies have suggested that females may be more likely to be exposed to non-smoking COPD risk factors, such as hormones, environmental or occupational exposures [38], and that there may be differences in cigarette smoking metabolism by sex [39]. Unfortunately, we were unable to adjust for these covariates due to lack of information. Moreover, our findings came from two separate cohorts, NHS and HPFS. Although these studies were designed consistently and conducted by the same institution, they may have underlying differences in the study populations beyond sex.

### 6-year COPD risk predictions

We estimated the 6-year risk of COPD incidence given various smoking scenarios using our model. The results show significant increases in COPD risk by longer smoking duration, higher smoking intensity, and older age. Our model can quantify the effect of various smoking levels on COPD risk. For example, if a 70-year-old female smoked 40 pack-years over 30 years, the probability of being diagnosed with COPD in next 6 years is 4.8%, which is 1.6% higher (percentage difference) compared to smoking 20 pack-years over the same duration. In contrast, if a 70-year-old female have smoked 40 pack-years over 20 years instead, the absolute 6-year risk would be reduced by 0.4%.

### Strengths and limitations

Strengths of our study include the availability of detailed high-quality longitudinal data on two large populations, which enabled us to examine the association between changes in smoking patterns over time and COPD incidence. Our study used lifetime smoking histories prior to COPD diagnosis; therefore, avoiding the temporal ambiguity that is usually present in cross-sectional studies. The model was developed accounting for time-dependent effects of smoking, reflecting that the association of smoking intensity and COPD incidence is not constant over time. Our model was well validated and has high discriminatory power, suggesting its potential to predict COPD risk accurately given individual’s smoking history.

Our study has some limitations. First, COPD incidence was defined by a self-report of physician-diagnosis. The absence of clinical confirmation may lead to an underestimation of the true COPD incidence. Barr et al. validated the self-reported COPD information in a subset of NHS cohort using participant’s medical information, including spirometry, chest radiographs, computed tomographies and physician diagnosis. Based on supplemental COPD and asthma questionnaires in the 1998 questionnaire, the self-reported COPD cases in NHS were classified into three categories: Definite, Probable, and Possible COPD. Barr et al. showed that 86%, 80% and 78% of self-reported definite, probable, and possible COPD cases, respectively, were confirmed by medical record review. If the analysis was restricted to only incident COPD cases, i.e., excluding prevalent COPD cases before 1988, the proportion of confirmed COPD cases increased to 90%, 84%, and 83% [40]. Therefore, our analysis based on self-reported COPD data in the NHS should provide an adequate assessment of COPD incidence. The HPFS study is consistent in design and methods with the NHS study, which suggest this is also the case for COPD incidence in HPFS. Second, our study population was predominantly Whites. Studies have suggested that Blacks may be more susceptible to COPD than Whites [41]. It is unclear whether this is due to other competing causes, difference in genetic susceptibility, or smoking behaviors [41,42]. Our model did not include race and socioeconomic factors, which may be associated with COPD risk [9]. The HPFS and NHS cohorts consist of health professionals and nurses, so their income levels may be similar across individuals, especially in the NHS cohort. Therefore, our results may not be extended to other races or socioeconomic groups. Moreover, our model only included age, sex and smoking-related information. There are other established COPD risk factors such as history of asthma [43,44], air pollution [45], secondhand smoking, occupational exposures, exposures to dust and fumes, socioeconomic status, childhood respiratory infections, lung function biomarkers, and other combustible tobacco use. Unfortunately, information was not available for any of these risk factors. Further studies extending the model to consider these and other covariates are needed. Research has shown significant variabilities of lung functions by smoking status, and those with lower lung function might be more susceptible to develop COPD [46]. Also, we excluded prevalent cases in 1998 from the analysis, which might bias our estimated COPD incidence in younger ages and for older individuals in 1998. This, nonetheless, makes our model and analysis more relevant to current patterns of smoking and COPD risk. Even with these limitations, by including detailed individual smoking histories, our model may be more generalizable than models based only on smoking status or pack-years. Finally, we validated the model internally using the same cohort with a split-sample approach; however, an external validation of the model may be needed to further demonstrate its applicability to other populations.

As illustrated, the model can calculate COPD incidence risk within a period of time as a function of age, sex, and individual smoking histories. Thus, the model could be used to calculate a score for risk stratification. Individuals at higher risk than a prescribed threshold could be recommended for specific risk reducing or early detection interventions, as is done for lung cancer and screening [40–42]. Further research to validate the performance of the model to identify individuals at high COPD risk in various settings and populations, and extensions to consider other relevant covariates, is needed before use in clinical practice.

### Conclusion and implications

In conclusion, we developed a COPD risk prediction model that incorporates individual time-varying smoking information. The model shows better discrimination accuracy for incidence of diagnosed COPD than models based on smoking status and pack-years only. The model might be useful in clinical settings to assess the COPD risk given an individual’s smoking history and identify patients at high risk of COPD. This model has also the potential to be integrated into micro-simulation models of smoking and health outcomes [26,47,48] to project the incidence and prevalence of COPD for the next decades as smoking patterns continue to evolve in the U.S.

## Supporting information

S1 Fig Flow chart for model building and validation datasets. (DOCX)

S2 Fig Relative risk of COPD incidence per one pack-year increase by age. (DOCX)

S1 Table Baseline characteristics of NHS (1976) and HPFS (1986) Cohorts in model building and validation datasets (mean and standard deviation). (DOCX)

S2 Table Characteristics of COPD cases (1998–2008) among NHS and HPFS cohorts in model building and validation datasets. (DOCX)

S3 Table Examples of 6-year absolute risk estimates for incidence of diagnosed COPD. Selected scenarios include current or former smokers at age 50, 60, 70 or 80, who have smoked either 20 pack-years or 40 pack-years. The smoking duration varies by 20, 30, or 40 years. These 6-year risk estimates were calculated with adjusting for other causes of mortality.
(DOCX)

S1 Text Calculation of the absolute risk of COPD diagnosis incidence. (DOCX)
