# Supplementary material for: Prediction of COPD risk accounting for time-varying smoking exposures
Source: PLoS One. 2021 Mar 10;16(3):e0248535. doi: 10.1371/journal.pone.0248535 (PMC7946316; doi:10.1371/journal.pone.0248535)
Supplement: S1 Fig — (DOCX) [file pone.0248535.s001.docx]

S1 Fig. Flow chart for model building and validation datasets.
