# Supplementary material for: Prediction of COPD risk accounting for time-varying smoking exposures
Source: PLoS One. 2021 Mar 10;16(3):e0248535. doi: 10.1371/journal.pone.0248535 (PMC7946316; doi:10.1371/journal.pone.0248535)
Supplement: S2 Fig — (DOCX) [file pone.0248535.s002.docx]

S2 Fig. Relative risk of COPD incidence per one pack-year increase by age.
