# Supplementary material for: Prediction of COPD risk accounting for time-varying smoking exposures
Source: PLoS One. 2021 Mar 10;16(3):e0248535. doi: 10.1371/journal.pone.0248535 (PMC7946316; doi:10.1371/journal.pone.0248535)
Supplement: S1 Table — (DOCX) [file pone.0248535.s003.docx]

S1 Table. Baseline Characteristics of NHS (1976) and HPFS (1986) Cohorts in model building and validation datasets (mean and standard deviation).

|  | Model building | | |  | Validation | |
| --- | --- | --- | --- | --- | --- | --- |
|  | HPFS (N=19,914)  Mean (SD) | | NHS (N=43,365)  Mean (SD) |  | HPFS (N=19,903)  Mean (SD) | NHS (N=43,346)  Mean (SD) |
| **Smoking intensity (pack-year)** | |  |  |  |  |  |
| Current smokers | | 30.99 (18.95) | 23.12 (14.43) |  | 30.64 (18.98) | 23.05 (14.35) |
| Former smokers | | 19.94 (17.29) | 11.86 (11.74) |  | 19.83 (17.39) | 11.64 (11.64) |
| **Smoking duration (year)** | |  |  |  |  |  |
| Current smokers | | 30.37 (10.82) | 23.40 (7.16) |  | 30.67 (11.19) | 23.45 (7.21) |
| Former smokers | | 20.29 (11.42) | 12.56 (7.88) |  | 20.08 (11.33) | 12.35 (7.77) |
| **Year-since-quit (year)** | |  |  |  |  |  |
| Former smokers | | 14.82 (10.78) | 11.82 (6.98) |  | 15.11 (10.85) | 11.94 (6.99) |
|  | |  |  |  |  |  |
| **Age at entry (year)** | | 53.28 (9.26) | 42.34 (7.12) |  | 53.32 (9.29) | 42.36 (7.12) |
| **COPD cases during the follow-up***  **N (%)** | | 700 (3.52) | 1,756 (4.05) |  | 758 (3.81) | 1,780 (4.11) |

* COPD incidence occurred during the follow-up (1998-2008) were included in the analysis.

HPFS=Health Professionals Follow-up Study; NHS=Nurses’ Health Study; SD=Standard deviation; COPD= Chronic Obstructive Pulmonary Disease; N=number of individuals in the data.
