# Supplementary material for: Prediction of COPD risk accounting for time-varying smoking exposures
Source: PLoS One. 2021 Mar 10;16(3):e0248535. doi: 10.1371/journal.pone.0248535 (PMC7946316; doi:10.1371/journal.pone.0248535)
Supplement: S2 Table — (DOCX) [file pone.0248535.s004.docx]

S2 Table. Characteristics of COPD cases (1998-2008) among NHS and HPFS cohorts in model building and validation datasets.

| Dataset | Smoking Status^1^ | N (%) | Diagnosis age  Median (IQR) | Smoking intensity^2^  Median (IQR) | Smoking duration^3^  Median (IQR) | Year-since-quit^4^  Median (IQR) |
| --- | --- | --- | --- | --- | --- | --- |
| Model building (HPFS) | Never | 165 (23.57) | 75.00 (16.00) | - | - | - |
|  | Former | 442 (63.14) | 75.00 (11.00) | 33.94 (33.00) | 31.00 (21.00) | 20.00 (24.00) |
|  | Current | 93 (13.29) | 69.00 (11.00) | 54.88 (34.62) | 47.00 (13.00) | - |
|  |  |  |  |  |  |  |
| Model building  (NHS) | Never | 457 (26.03) | 71.00 (10.00) | - | - | - |
|  | Former | 989 (56.32) | 72.00 (9.00) | 36.00 (34.00) | 34.00 (22.00) | 19.00 (21.00) |
|  | Current | 310 (17.65) | 69.00 (9.00) | 57.25 (28.38) | 50.00 (10.00) | - |
|  |  |  |  |  |  |  |
| Validation  (HPFS) | Never | 192 (25.33) | 74.00 (12.50) | - | - | - |
|  | Former | 465 (61.35) | 76.00 (10.00) | 33.50 (31.50) | 33.00 (20.00) | 20.00 (20.00) |
|  | Current | 101 (13.32) | 70.00 (12.00) | 48.50 (34.12) | 48.00 (14.00) | - |
|  |  |  |  |  |  |  |
| Validation  (NHS) | Never | 491 (27.58) | 72.00 (11.50) | - | - | - |
|  | Former | 985 (55.34) | 72.00 (10.00) | 34.00 (34.25) | 34.00 (22.00) | 17.00 (22.00) |
|  | Current | 304 (17.08) | 69.00 (10.00) | 58.00 (30.12) | 50.50 (11.00) | - |

^1^ Smoking status at the time of COPD diagnosis; ^2^ Cumulative smoking pack-years at the time of COPD diagnosis; ^3^ Cumulative smoking duration in years at the time of COPD diagnosis; ^4^ Years since quit in years for former smokers at the time of COPD diagnosis. HPFS=Health Professionals Follow-up Study; NHS=Nurses’ Health Study; IQR= Interquartile range; COPD= Chronic Obstructive Pulmonary Disease; N=number of individuals in the data.
