# Supplementary material for: Prediction of COPD risk accounting for time-varying smoking exposures
Source: PLoS One. 2021 Mar 10;16(3):e0248535. doi: 10.1371/journal.pone.0248535 (PMC7946316; doi:10.1371/journal.pone.0248535)
Supplement: S3 Table — Selected scenarios include current or former smokers at age 50, 60, 70 or 80, who have smoked either 20 pack-years or 40 pack-years. The smoking duration varies by 20, 30, or 40 years. These 6-year risk estimates were calculated with adjusting for other causes of mortality. (DOCX) [file pone.0248535.s005.docx]

S3 Table. Examples of 6-year absolute risk estimates for incidence of diagnosed COPD. Selected scenarios include current or former smokers at age 50, 60, 70 or 80, who have smoked either 20 pack-years or 40 pack-years. The smoking duration varies by 20, 30, or 40 years. These 6-year risk estimates were calculated with adjusting for other causes of mortality.

|  |  | **Smoking Duration (years)** | | | | | | | |
| --- | --- | --- | --- | --- | --- | --- | --- | --- | --- |
|  |  | **20 years** | |  | **30 years** | |  | **40 years** | |
| **Scenario** | **Age (year)** | **Current smokers**  **Risk % (95% CI)** | **Former smokers***  **Risk % (95% CI)** |  | **Current smokers**  **Risk % (95% CI)** | **Former smokers***  **Risk % (95% CI)** |  | **Current smokers**  **Risk % (95% CI)** | **Former smokers***  **Risk % (95% CI)** |
| **Females: 20 PY** | 50 | 0.8 (0.7 to 0.8) | 0.2 (0.2 to 0.2) |  | 0.9 (0.8 to 0.9) | 0.3 (0.3 to 0.3) |  | 1.0 (0.9 to 1.0) | 0.3 (0.3 to 0.3) |
|  | 60 | 1.4 (1.3 to 1.4) | 0.6 (0.6 to 0.6) |  | 1.6 (1.5 to1.6) | 0.7 (0.7 to 0.8) |  | 1.8 (1.7 to1.8) | 0.9 (0.8 to 0.9) |
|  | 70 | 1.8 (1.8 to 1.8) | 1.2 (1.2 to 1.3) |  | 2.1 (2.0 to 2.1) | 1.5 (1.4 to 1.5) |  | 2.4 (2.3 to 2.4) | 1.7 (1.6 to 1.7) |
|  | 80 | 1.5 (1.5 to 1.5) | 1.4 (1.4 to 1.4) |  | 1.7 (1.6 to 1.7) | 1.6 (1.6 to 1.7) |  | 1.9 (1.9 to 2.0) | 1.9 (1.9 to 1.9) |
| **Females: 40 PY** | 50 | 1.5 (1.4 to 1.5) | 0.4 (0.4 to 0.4) |  | 1.6 (1.5 to 1.7) | 0.5 (0.5 to 0.6) |  | 1.8 (1.7 to 1.9) | 0.5 (0.5 to 0.6) |
|  | 60 | 2.4 (2.3 to 2.4) | 1.0 (1.0 to 1.0) |  | 2.6 (2.5 to 2.7) | 1.2 (1.2 to 1.2) |  | 3.0 (2.9 to 3.0) | 1.4 (1.4 to 1.4) |
|  | 70 | 2.8 (2.7 to 2.9) | 1.8 (1.8 to 1.9) |  | 3.1 (3.0 to 3.2) | 2.1 (2.1 to 2.2) |  | 3.5 (3.4 to 3.5) | 2.5 (2.4 to 2.5) |
|  | 80 | 2.0 (2.0 to 2.1) | 1.9 (1.8 to 1.9) |  | 2.2 (2.2 to 2.3) | 2.1 (2.1 to 2.2) |  | 2.5 (2.5 to 2.6) | 2.5 (2.4 to 2.5) |
| **Males: 20 PY** | 50 | 0.3 (0.3 to 0.3) | 0.2 (0.2 to 0.2) |  | 0.3 (0.3 to 0.4) | 0.3 (0.3 to 0.3) |  | 0.4 (0.4 to 0.4) | 0.4 (0.3 to 0.4) |
|  | 60 | 0.7 (0.7 to 0.7) | 0.6 (0.6 to 0.6) |  | 0.9 (0.8 to 0.9) | 0.8 (0.7 to 0.8) |  | 1.1 (1.0 to1.1) | 0.9 (0.9 to 1.0) |
|  | 70 | 1.2 (1.2 to 1.3) | 1.1 (1.0 to 1.1) |  | 1.5 (1.4 to 1.6) | 1.3 (1.2 to 1.4) |  | 1.8 (1.7 to 1.9) | 1.6 (1.5 to 1.7) |
|  | 80 | 1.1 (1.1 to 1.1) | 1.0 (0.9 to 1.0) |  | 1.3 (1.3 to 1.4) | 1.2 (1.1 to 1.3) |  | 1.6 (1.5 to 1.7) | 1.5 (1.3 to 1.5) |
| **Males: 40 PY** | 50 | 0.6 (0.5 to 0.6) | 0.4 (0.4 to 0.4) |  | 0.6 (0.6 to 0.7) | 0.5 (0.5 to 0.5) |  | 0.8 (0.7 to 0.8) | 0.6 (0.6 to 0.7) |
|  | 60 | 1.3 (1.2 to 1.3) | 1.0 (0.9 to 1.0) |  | 1.5 (1.4 to 1.6) | 1.2 (1.2 to 1.3) |  | 1.8 (1.6 to 1.9) | 1.5 (1.4 to 1.6) |
|  | 70 | 1.9 (1.8 to 2.0) | 1.6 (1.5 to 1.6) |  | 2.3 (2.1 to 2.4) | 1.9 (1.8 to 2.0) |  | 2.7 (2.5 to 2.9) | 2.4 (2.2 to 2.5) |
|  | 80 | 1.5 (1.4 to 1.6) | 1.3 (1.2 to 1.3) |  | 1.8 (1.7 to 1.9) | 1.6 (1.5 to 1.6) |  | 2.1 (2.0 to 2.2) | 1.9 (1.8 to 2.0) |

*Former smokers stop smoking at the corresponding age 50, 60, 70 or 80; PY=Pack-years; CI=Confidence interval; The 95% CIs were calculated using the Bootstrap method with 100 iterations.
