# Supplementary material for: Prediction of COPD risk accounting for time-varying smoking exposures
Source: PLoS One. 2021 Mar 10;16(3):e0248535. doi: 10.1371/journal.pone.0248535 (PMC7946316; doi:10.1371/journal.pone.0248535)
Supplement: S1 Text — (DOCX) [file pone.0248535.s006.docx]

S1 Text. Calculation of the absolute risk of COPD diagnosis incidence

We estimated the 6-year absolute risk of incidence of diagnosed COPD and corresponding 95% CIs for a given individual's smoking history as described below. Let $T_{1}, T_{2},$and $T_{e}$be the age of COPD diagnosed incidence, age of dying from other causes, and age at study entry, respectively. The probability of being diagnosed with COPD in the next 6 years is calculated as following:

$$P(T_{e}<T_{1}\leq T_{e}+6)=\int_{T_{e}}^{T_{e}+6} h_{1}(u)S_{1}(u)du,$$

where $h_{1}\left( u \right)$ is the hazard function for the COPD incidence and $S_{1}\left( u \right)$ the corresponding survival function, which can be obtained by the following equation: $S_{1}\left( u \right)=e^{-\int_{0}^{u} h_{1}\left( s \right)ds}.$

Similarly, we also calculated the probability of being diagnosed with COPD in the next 6 years with accounting for other causes of death:

$$P(T_{e}<T_{1}\leq T_{e}+6, T_{1}<T_{2})=\int_{T_{e}}^{T_{e}+6} h_{1}(u)S_{1}(u)S_{2}(u)du,$$

where $\boldsymbol{S}_{\boldsymbol{2}}\boldsymbol{(u)}$ is the survival function for the death from causes other than COPD, which was obtained from the US life-table stratified by smoking status. The 95% CIs were calculated using the Bootstrap method with 100 iterations.
